# A Practical Treatise on the Diseases of the Eye

**Published:** 1831

**Authors:** 


A PRACTICAL TREATISE ON THE DISEASES OF THE EYE. By *William Mackenzie*, Lecturer on the Eye in the University of Glasgow, and one of the Surgeons to the Glasgow Eye Infirmary. 8vo. pp. 861, London, 1830.

[Second Analytical Article.]

IN a former article we made our readers acquainted with Mr. Mackenzie's sentiments respecting inflammation of the conjunctiva, sclerotica, and cornea. In considering the remainder of the ophthalmiæ, or inflammatory diseases of the eye, many subjects press on our attention. We must view the iris, the choroid, the retina, the capsules of the lens and vitreous humor in a state of inflammation, and then we must look to the traumatic ophthalmiæ, the compound, and the intermittent. A knowledge of these affections is of no mean importance to the general practitioner, as well as to the surgeon and physician, indeed no man, whatever may be his designation or rank in the profession, can practice it with credit, or with justice to the community, unless he be well informed in these matters. In acute inflammation of the more delicate textures of the eye, a blunder from ignorance, or delay from doubt, may make the difference between the continuance of vision or the utter loss of it. We cannot too strongly impress upon the minds of students, the paramount necessity of making themselves masters of this department, at least, of ophthalmic science. Without much practice it is impossible for any man to perform in a satisfactory manner the more delicate operations on this delicate organ, indeed, he is hardly justified in attempting them. But all may instruct themselves in the principles of treatment of those diseases of the eye which all must have an opportunity of witnessing, and the management of which most men will find themselves called on to undertake.

### IRITIS.

The discrimination of this disease has formed a highly important addition to our knowledge. It often exists independent of inflammation in the other textures of the eye, and its insidiousness and danger render it a matter deserving of great attention. There is always a degree of sclerotic inflammation attending it, and the anterior hemisphere of the capsule of the lens is more or less affected, whilst too often the inflammatory action extends to the choroid and retina. The iris, however, is the focus of the disease, which commences on its pupillary margin, and thence spreads to the parts alluded to. The sclerotic inflammation appears to be sympathetic. That the iris only is in many cases affected seems proved by the fact that an artificial pupil often restores vision, when the natural pupil has been closed by inflammation. Before proceeding to treat of the varieties of iritis, we are presented with a section on the affection in general.

"*Symptoms.* There are certain symptoms which characterize inflammation of the iris, from whatever cause it proceeds.

1. Zonular sclerotitis; fine hair-like vessels running in radii towards the edge of the cornea.
2. Discolouration of the iris. If naturally blue, it becomes greenish; if dark-coloured, reddish.
3. Contraction, irregularity and immobility of the pupil.
4. Effusion of coagulable lymph into the pupil and posterior chamber, and occasionally into the anterior.
5. Adhesions of the iris, and especially of its pupillary edge, to the capsule of the lens; in some rare cases, to the cornea.
6. Dimness of sight, and sometimes almost total blindness.
7. Pain in the eye, and nocturnal circumorbital pain.

In every case of iritis, a sufficient number of these symptoms will be met with, to enable the observer to decide on the seat of the disease which is before him. All of them are by no means invariably present. We sometimes find, for instance, a dilated pupil in iritis, probably from the coexistence of amaurosis; and in some otherwise well-marked cases, there is not the slightest circumorbital pain. The disease may also exist in a very marked manner, without any effusion of lymph, or preternatural adhesions of the iris, these being part of the changes which take place only in the second stage of iritis." 423.

*Causes.* These are various. Exposure to atmospheric changes, especially transitions from heat to cold—syphilis and syphiloid diseases—struma, the iritis being usually secondary to corneitis—gout—and local injuries, are the causes which are best ascertained. Iritis has also been described as arising from mercury, and from typhus fever.

*Prognosis.* Iritis may exist in different degrees of severity, and on these the chance of cure must entirely hinge. *In the first degree*—the vascularity in front of the sclerotica is very slight, and existing sometimes only in one or more points, or behind the upper lid, where it might not be discovered—the inner ring of the iris is slightly discoloured—the pupil is of medium-size, but wants its usual clean sharp edge, and may be irregular or misshapen, it is hazy, and its motions are limited and slow—vision is slightly obscure and confused—there is no severe pain and scarcely any intolerance of light. This iritis may exist for many weeks, and yet, by proper treatment be completely overcome. *In the second degree*—the external inflammation is considerable and obvious, the sclerotica presenting round the cornea innumerable ramifications of vessels, forming a complete radiated zone—these vessels seem to terminate abruptly, as if sinking through the sclerotica, and never advance into or over the cornea—the inner, and partially the outer ring of the iris are discoloured from its injection with blood, or perhaps from effusion of lymph into its substance, a change of colour which is apt to be permanent—the anterior surface of the iris appears dull, puckered, and swollen, especially near the pupil, which is retracted towards the lens—the pupil is contracted, irregular, motionless, and filled with coagulable lymph, looking like half-boiled white of egg—vision is greatly impaired—epiphora and intolerance of light considerable—the pain of the eye pretty constant, and attended during the night with circumorbital hemicrania—and there are symptoms of inflammatory fever.

"From such a state of the eye, recovery to a certain extent may take place even without any very methodical treatment. By the use of proper remedies, the inflammation will gradually be subdued, and the effused lymph be absorbed; the contracted pupil will expand, though probably never so completely as to regain its natural size or mobility, and a tolerably fair state of vision will ultimately be recovered. As the symptoms yield, whitish threads of organized lymph will become evident, binding at different points the edge of the pupil to the capsule of the lens. These adhesions are capable of being elongated in time, but never disappear entirely, and necessarily impede the functions of the iris. In other cases, the whole of the edge of the pupil is fringed with lymph, firmly gluing it to the capsule, the centre of which may also be left opaque from lymphatic deposition, in which case the patient sees only through the imperfectly transparent ring left between the central opacity of the capsule, and the fringed edge of the pupil. It sometimes happens in this degree of the disease, that the posterior surface of the annulus minor, which is covered with pigmentum nigrum, having been glued by lymph to the anterior capsule, the proper substance of the iris, as the inflammation subsides, regains, in a considerable measure, its power, and the pupil is enlarged, while the pigmentum nigrum remains adherent to the capsule, and is seen of a black colour fringing the edge of the pupil, and constituting a variety of what has been called *cataracta pigmentosa*." 425.

In the *third* degree of iritis, the surface of the eye is much more intensely inflamed.

"The conjunctiva may be so much so, as completely to mask for a time the zonular redness of the sclerotica. Both the annulus minor and major of the iris lose their natural colour. The anterior surface of the iris is puckered, swoln, and bolstered forward so as to approach the cornea, except its pupillary edge, which is retracted towards the capsule of the lens. Red vessels and spots of blood may sometimes be discovered on the surface of the iris, and still more frequently in the lymph which occupies the contracted pupil. On the surface of the iris, one or more minute elevations of a yellowish colour make their appearance, which in some cases are merely spots of effused lymph, but in others prove small abscesses. Pus, discharged from these, with lymph, and blood, occupy the anterior chamber. The cornea becomes turbid, so as to resemble a piece of glass which has been breathed upon, and in some cases is dotted over with minute brownish spots. Vision is completely, and, in general, permanently lost. Flashes of light in the eye are frequently perceived by the patient, proving that the disorganization is extending to the choroid and retina. There is great intolerance of light, and copious lachrymation. The pain of the eye which attends this third degree of iritis, is in general constant and excruciating, and attended with severe nocturnal pain in the eyebrow and round the orbit. When the case is attended by severe and unmitigated pain, especially in syphilitic cases, there is reason to dread the most serious changes in the eye, even abscess of the anterior chamber, extenuation of the sclerotica, and protrusion of the choroid immediately behind the cornea, disorganization of the vitreous humour, and ultimately atrophy of the eyeball." 426.

In this degree of iritis the prognosis must always be unfavourable, for under the most prompt and proper treatment, any thing near to a perfect recovery of the eye never takes place. The inflammation will subside, the effused lymph and pus will be taken up from the anterior chamber, but the pupil will never become entirely clear, nor regain almost any degree of motion. Sometimes the iris is so contracted that no vestige of pupil can be discovered, most frequently it remains of the size of a pin-hole, through which a considerable share of vision may be enjoyed, though in most instances this is not the case; and in many, the choroid and retina being diseased, not even an artificial pupil can restore vision.

The distinction of acute and chronic iritis is of great importance. The former takes place in robust individuals, from powerful causes, and more especially if the case has at first been neglected. There are bright external redness, great distention of vessels, rapid and general change of colour in the iris, contraction of the pupil, effusion of lymph, dulness of the cornea, loss of sight, agonizing pain of the eye, severe headache, and considerable fever, restlessness and want of sleep. In a few days vision is irreparably lost. The chronic iritis may arise so imperceptibly, and proceed so slowly that the patient has been known to find accidentally that the vision of the eye is gone. Extension of the inflammation to the rest of the organ occurs more readily in acute cases, yet may equally take place in the chronic. The prognosis must be drawn from the time the disease has lasted, its cause, and its visible effects. In the acute affection irreparable injury may be done in a few days, or, if only ordinarily severe, a fortnight, three weeks, or a month may elapse without serious mischief; in the most chronic form recovery may be expected after a still longer period.

*Sequelæ of the Disease.* The most striking are the changes which the pupil undergoes. The inflammatory symptoms, however severe, begin after a time to abate; if pus and blood have been effused they are gradually absorbed: if an abscess has formed on the iris, the shreds of the cyst at length disappear: and the anterior chamber regains its transparency. In many cases the iris remains permanently expanded, with its motions annihilated. The greater circle may in some measure resume its natural colour, but the lesser does not. The puckered appearance of the iris remains, the pupil is almost closed, and filled by an ash-coloured membrane, the power of vision is entirely lost. This is called *atresia iridis completa.* The condition of the iris is not always so bad. Perhaps there has been no abscess, nor any great quantity of lymph; the iris is left considerably expanded, but still possesses some mobility, and possibly its natural colour may be almost restored. Though the pupil is contracted below its medium size, the coagulable lymph occupying it is reduced to the state of fine pseudo-membrane, mostly opaque in the centre, but somewhat transparent, perhaps reticulated at the edge. The pupillary margin of the iris does not adhere to it all round, but only at some points, rendering the pupil very irregular, impairing vision but not destroying it, constituting *atresia iridis incompleta.* In a third set of cases only a part of the iris has been inflamed, and a mere thread of opaque matter remains in the otherwise transparent pupil. By this only a single point of the margin of the pupil is kept fixed, and this is termed *atresia iridis partialis.*

*Diagnosis.* The ophthalmiæ with which iritis is apt to be confounded, are rheumatic and catarrho-rheumatic ophthalmiæ, corneitis, aquo-capsulitis, inflammation of the crystalline capsule, and retinitis. Of the three first we have already given a description in a preceding article, and of the three last we shall treat presently; consequently we need not detain our readers with the diagnostic marks of these diseases.

*General Treatment of Iritis.* The chief indications are, to subdue the inflammation; to prevent the effusion of lymph, or promote its absorption; to preserve the pupil entire, or, if contracted, to dilate it; to assuage the pain. Blood-letting must not be neglected; if the patient is robust it must be vigorously employed. General bleeding must be resorted to till the constitutional irritation is abated, and then leeches may be freely applied round the eye every second or third day. Purging, diuretics, the antiphlogistic regimen, and shading both eyes from the light are powerful auxiliaries. Antimony and other nauseants are doubly useful; opiates imperatively demanded by the severe nocturnal pain; mercury a most valuable remedy; turpentine has been recommended lately by Mr. Carmichael; blisters behind the ears are of service after sufficient depletion. Belladonna, in the first degree of iritis, speedily expands the pupil; in the second and third it has no effect till the inflammation has been considerably subdued. It ought to be employed in every case and in all stages of the disease, the extract being smeared on the brow and upper eyelid every evening. Even in neglected cases, where the pupil has become almost obliterated, the continued use of belladonna, for many months, is sometimes attended with gradual dilatation, and improvement in vision. In short, after taking away blood, Mr. Mackenzie would never hesitate to apply belladonna. Such are the general remedies for iritis; its particular varieties require particular modifications of treatment.

### I.—RHEUMATIC IRITIS.

Attending on the rheumatic and catarrho-rheumatic ophthalmiæ, there is in general a certain degree of iritis. But there is a third set of cases, arising, like the preceding, from atmospheric changes, in which the iris is chiefly affected, and the attack sudden, as in other diseases caused by external influences. Not unfrequently both eyes are affected with nearly equal severity; in other cases only one eye is inflamed, or one much more severely than the other.

*Local Symptoms.* At the very commencement of the disease changes occur indicative of the peculiar seat of the inflammation; they uniformly commence on the edge of the pupil, and gradually extend towards the ciliary circumference of the iris. The pupil is first of all contracted, the motions of the iris impeded, and the pupillary opening deprived of its natural bright black colour. Then the colour of the iris changes; first the lesser circle becomes of a darker hue, and afterwards the greater grows green, if it had formerly been greyish or blue; reddish, if it had been brown or black. This is a never failing index of inflammation of the iris, and is apt to continue after the other symptoms of iritis have been subdued. As soon as it has taken place to a considerable degree in the greater circle, the iris swells and projects towards the cornea, while the pupillary margin is somewhat thickened, and is turned back towards the capsule of the lens. The accompanying redness is not great, and is at first confined to the sclerotica, in which a number of minute red vessels are seen, running in lines towards the cornea. By and bye the redness increases, and partly arises from vessels developed in the conjunctiva; the vascularity is greatest round the cornea, and fades away towards the folds of the conjunctiva. There is pain in the eye, severe and pulsative in many cases, and increased on motion of the organ; pain in the eyebrow; and circum-orbital nocturnal pain.

If the disease is not checked, the pupil loses its circular form, and becomes irregular and greyish. Examined through a magnifying glass its appearance is seen to be produced by a substance very like a cobweb, a delicate flake of coagulable lymph. Into this the dentations of the irregular pupillary margin of the iris seem to shoot, and, at this point, adhesions are afterwards apt to be established between the iris and capsule. Owing to these adhesions the patient complains of being able to see only one side or part of an object.

The effusion of lymph into the pupil continues to increase, and, taking place behind the iris, adhesions are formed between the uvea and capsule of the lens. The quantity of lymph is sometimes so great, as to fall down like curd into the anterior chamber. The morbid sensibility to light, which prevailed in the early stage of the disease, is now diminished; the powers of vision grow more and more limited, and at length little more than perception of light remains. Not unfrequently, the lymph occupying the contracted pupil gives rise to the sensation of a black spot, or fly, or several such spots, placed at some distance before the eye, and partially intercepting the view of objects placed before or to one side of the patient.

As the disease goes on the cornea loses some of its brilliancy, and sometimes striking changes take place on the anterior surface of the iris. Spots of lymph occasionally form upon it; at other times lymph appears deposited in its substance, its fibres getting collected into bundles, and its surface acquiring a peculiar plaited or puckered aspect. In some cases one or more small yellowish-red elevations form on the anterior surface of the iris, commonly about the union of its greater and lesser circles; the elevation gradually increases, is distinctly seen to be a cyst containing pus, bursts, and discharges its contents into the anterior chamber, thus giving rise to spurious hypopium, a small quantity of blood being extravasated into that cavity at the same time. Such is the general history of a neglected case of rheumatic iritis, though many varieties of severity are met with. The inflammation will at last subside, even though no remedies are employed, but then the vision is generally lost.

*Constitutional Symptoms.* This may attack an individual who has never suffered from rheumatism elsewhere. Not unfrequently the subjects have long been subject to other rheumatic affections, though Mr. Mackenzie has never seen this affection arise from any thing but a fresh exposure to cold. During the attack there is pyrexia, frequently constipation, occasionally nausea.

*Causes.* They are the same with those producing rheumatic ophthalmia. Some persons suffer from one or more attacks of this disease every year, till at length vision is destroyed. This iritis frequently occurs during or after the use of mercury.

*Complication with Amaurosis.* This is by no means rare, and is particularly frequent after typhus fever, a disease extremely apt to leave the retina more or less insensible, and the pupil dilated.

"Mr. Wallace has described the complication of amaurosis with iritis after typhus fever, as presenting two distinct stages. During the first stage, there exist amaurotic symptoms alone; in the second, symptoms of inflammation are superadded. The length of time that the amaurotic symptoms continue, before the occurrence of any visible appearance of inflammation, is extremely uncertain, as also the period after fever at which the amaurotic symptoms commence. On many occasions, the amaurotic symptoms, particularly a slight dimness of vision, with muscæ volitantes, have commenced at or even before the time of convalescence from fever, and yet the inflammatory stage has not supervened for weeks or even months; while on other occasions the dimness of vision has not commenced for several days, weeks, or even months, after the febrile attack, and has then been immediately followed by the symptoms of inflammation. Mr. W. never saw a case in which, upon strict inquiry, amaurotic symptoms, more or less strongly marked, had not preceded the inflammatory symptoms. He also observed that the inflammatory symptoms uniformly subsided a longer or shorter time before the amaurotic symptoms disappeared, and often before they had even diminished in severity."* 434.

*Treatment.* Repeated venesection is almost always necessary, followed by the free application of leeches round the eye; the degree of depletion must be regulated by the symptoms. As soon as the mouth is affected by mercury we observe the most marked abatement of the symptoms, but the slightest exposure to cold will induce a relapse with ten fold severity. The patient must confine himself within doors, if the case be severe he must keep his bed; the room should be darkened, there should be a moderate fire in it, if in Winter, a band of flannel should be constantly worn round the head, and several folds of linen over the eye. In pauper patients, out of hospital, it becomes a difficult question whether we can venture on mercury at all. Turpentine may be tried; rest and the antiphlogistic regimen must be strictly enjoined. If calomel is given, it is combined with opium, at bed-time. If we do not give calomel, a powerful opiate should be given every night. Friction of the head with warm laudanum, or with mercurial ointment containing opium, is to be employed, but if these means fail to prevent the nocturnal pain, fomenting the eye-lids and parts around with

* "Medico-Chirurgical Transactions, Vol. xiv. p. 294. London, 1828."

flannel cloths wrung out of poppy decoction is beneficial, the parts being dried immediately after the fomentation, and the linen compress, previously heated, being then replaced. Moderate purgatives and diuretics are of service. Diaphoretics require caution.

Cinchona is undoubtedly useful in certain cases, but, as might well be expected, it is not so in all. It is chiefly serviceable in the combination of amaurosis with iritis after typhus, but still Mr. Mackenzie is of opinion that the treatment of this is best commenced with depletion and mercury. The acute symptoms having been subdued by these means, cinchona or quina are useful, not only in this species, but in ordinary rheumatic iritis. In the combined iritis and amaurosis after typhus, our author suspects some lingering congestion of the head, for in a case which he lately treated immediate relief was afforded by the abstraction of thirty ounces of blood from the arm, after various other remedies had failed. Blisters are more serviceable in rheumatic iritis than in any other. The laudanum with which the head is rubbed for the nocturnal pain may be mixed with an equal quantity of tincture of cantharides. Belladonna should be freely applied morning and evening. The vinum opii is useful in the decline of the disease; any other application is injurious. After all, prevention is better than cure. Let those subject to the disease avoid its exciting causes. Sea-bathing in Summer sometimes prevents relapses, and removal to a southern climate during the Winter may save a patient from his usual attack.

The next section, which treats of Syphilitic Iritis, we shall pass without further notice, having lately dedicated so much space to this ophthalmia in our analysis of Mr. Lawrence's book. We have no such obligation to prevent us from touching on—

### II. Pseudo-syphilitic Iritis.

The ecthyma cachecticum of Bateman is apt to be confounded, says Mr. Mackenzie, with true syphilis, and no doubt it occasionally affects the iris in a manner closely resembling syphilitic iritis. Of the ecthyma cachecticum we need not give a description; suffice it to say that, according to Dr. Bateman, it is quite curable by vegetable tonics, and that the administration of mercury is neither necessary to, nor appears to accelerate recovery. Dr. Monteath affirms that, for several years he treated cases of iritis successfully with mercury, believing them to be syphilitic, but that seeing such disease evidently decline, after two or three weeks' continuance without a grain of mercury being taken, he felt satisfied that the cases were not syphilitic. In these cases the small circle of the iris and the border of the pupil are often studded with the small reddish-yellow papulæ or pustules, so characteristic of the venereal iritis. Mr. Mackenzie recommends, notwithstanding, an alterative course of mercury, aided by sarsaparilla, local bleeding, blisters, belladonna, mild diet, &c. Turpentine is worthy of a trial. Among the pseudo-syphilitic varieties of iritis, Mr. Mackenzie includes that which occasionally follows gonorrhœa. Such are the observations on pseudo-syphilitic iritis, and if they amount to little or nothing, they are about as much to the purpose as any other descriptions of what is called pseudo-syphilis.

### III. SCROFULOUS IRITIS.

The iris is rarely the seat of primary scrofulous inflammation, but after scrofulous inflammation of the cornea, it is by no means uncommon as a secondary affection. In a scrofulous subject, however, cold occasionally brings on a mixed ophthalmia, partly phlyctenular, partly iritic, or at least the latter so speedily supervenes to the former, that we may regard it as primary scrofulous iritis. Whenever iritis is observed in a very young person, struma may be suspected as the predisposing cause, the other varieties being rare in childhood. The treatment must consist in the exhibition of calomel and opium, in addition to the remedies required for strumous ophthalmia. The pupil should also be kept under the influence of belladonna. The same plan must be followed in secondary iritis, following strumous corneitis. The difficulty of ascertaining the state of the iris through the inflamed cornea has already been adverted to, and many of the symptoms are equivocal. When the opacity of the cornea is not very great, we may discern at least the size and mobility of the pupil, and if it is contracted, irregular, and motionless, severe iritis has been present. In many cases, by concentrating the light upon the cornea through a double convex lens, we may even observe the discoloration of the iris, and the whitish cobweb of effused lymph occupying the pupil. Neglected cases of this disease are often met with, in which vision has become almost extinct. In such cases there is a remarkable degree of softness or bogginess both of the cornea and sclerotica when pressed by the finger. This is, in Mr. Mackenzie's opinion, a very unfavourable sign, denoting a disorganization of the vitreous humour, always attended by a considerable degree of iritis.

In treating this disease we must attempt, by mercury and belladonna, to counteract the contracted state of the pupil and effusion of lymph from the iris. Mercury must be given with more than ordinary caution and patience, the gums requiring in the first instance to be decidedly affected, after which repeated gentle courses of the medicine will be necessary, the system being supported in the intervals by nourishing diet and tonics. Turpentine has not been tried. We must beware of stimulants to the cornea, so long as any iritis is present, or we may bring on an irreparable degree of irritation.

### IV. ARTHRITIC IRITIS.

Mr. Mackenzie is not quite satisfied of this iritis being of so purely a gouty character as the German ophthalmologists maintain. However, gout not being so common a disease among the poorer classes of this country, and particularly we should imagine of Scotland, as in the wine countries of the Continent, Mr. Mackenzie does not feel justified in pronouncing a confident opinion on the subject. Arthritic iritis originates in two ways. In one case, it is the primary and sole affection of the eye; in the other, a gouty person being affected with some gouty ophthalmia, this degenerates into iritis.

*Symptoms.* The general symptoms of iritis are present in this species, but so modified as to afford ground for a ready diagnosis. Both the conjunctiva and sclerotica are loaded with enlarged vessels, the redness of a purplish hue, and, what is strongly insisted on as a diagnostic mark, the inflamed vessels stopping abruptly before reaching the edge of the cornea by a narrow ring of a bluish-white colour. This ring sometimes does not occur, particularly at the commencement of the disease, all round the cornea, but only at its temporal and nasal sides. The visible arteries of the eye shew from the very first a strong disposition to be varicose, and at length are so dilated as to form another characteristic symptom. The sclerotica becomes of a dirty greyish-violet colour. The iris and pupil exhibit changes varying in two different habits of body. In those of a meagre irritable habit, the pupil contracts, is filled with effused lymph, and becomes adherent to the capsule, as in the other species of iritis. The only characteristic symptom, besides the white ring round the cornea, is a varicose state of the vessels of the iris, which may be observed ramifying on the surface of that membrane, or forming a vascular wreath within the verge of the contracted pupil. Before the inflammation arrives at this stage there is always general fever. The eye, if left to itself, does not suppurate, but its contents begin to be absorbed, and at last its volume is extremely diminished. In those of a gross habit of body, with little sensibility, the iris, instead of expanding, contracts remarkably, a sign of attending amaurosis, and loses its motion and natural black colour. The pupil is not always uniformly dilated; not unfrequently the iris contracts more towards the temporal and nasal sides of the eye, so that the pupil assumes an oval shape, indeed the iris sometimes becomes so narrow on these sides, as nearly to disappear. There is no effusion of lymph, nor any formation of pus. Behind the enlarged pupil is perceived the greyish green glaucomatous reflection, depending on absorption of the pigmentum nigrum, with dissolution of the vitreous humour, and occasionally accompanied by discolouration of the lens. After a time the lens has lost its transparency, and assumed an opaque sea-green colour, it swells considerably, and projects through the pupil into the anterior chamber. The iris lying on it seems much altered, looking soft, and as if it had undergone a degree of maceration. The varicose state of the vessels of the conjunctiva increases, while those of the choroid, becoming similarly affected, form bluish knots, which shine through the sclerotica. The anterior part of this tunic being extenuated by the pressure of the morbid parts within, a dark ring shines through it, exactly occupying the situation of the corpus ciliare. Vision is now totally gone. The inflammatory symptoms begin to decrease, absorption of the eyeball follows, and in either variety of this disease, if both eyes are not simultaneously attacked, the same process invades the one after the other, till both are destroyed.

"*Pain.* It sometimes happens that before any other signs of arthritic ophthalmia make their appearance, the patient is troubled with peculiar tingling sensations about the eye, and a feeling of creeping over the skin of the face. The eye and the orbit soon become the seat of racking pain, extending to the temple, and shooting down into the jaws. During the progress of the changes of structure above detailed, the attacks of pain are regular and very severe, greatly aggravated in general towards midnight, but in some cases suffering little abatement at any period of the twenty-four hours. The patient is warned of their approach, by a stinging sensation all round the eye, followed by an increased flow of tears; after which, the pain sets in, and becomes, in many instances, so extremely violent, that the patient is forced to writhe under it, and to utter the most piercing cries of distress.

*Secretion from eyelids.* The epiphora which attends arthritic inflammation of the iris, leads to frequent opening and shutting of the eyelids, by means of which there is forced out from between them, a peculiar white frothy matter, which Beer regarded as diagnostic of arthritic ophthalmia, and which is easily distinguished from any of the ordinary secretions of the conjunctiva or Meibomian follicles. On examining this foam or froth, it appears to consist of extremely minute globules of watery fluid." 453.

*Constitutional Symptoms.* The subjects of arthritic iritis have generally suffered more from the symptoms of irregular than of regular gout. They generally present the marks of the gouty constitution, and are liable to dyspeptic symptoms, headaches, irregular bowels, pains in various parts, eruptions of suppurating tubercles on the face, &c. These patients have generally adopted an erroneous plan of diet, and especially indulged in alcoholic fluids and tobacco. The prognosis is more unfavorable than in any other species of iritis. A first attack may continue for months, and though it then yield, a relapse is always to be dreaded. Besides this, the disease has a strong and most unfortunate tendency to affect the choroid, retina, and humors, so that, though the attack be several times iritic, the rest of the eye-ball becomes at length implicated and destroyed.

*Treatment.* The indications are, to remove the inflammation—subdue the pain—prevent relapses. The inflammation is of an unsound, peculiar, and constitutional nature. General bleeding is seldom advisable, and even cupping and leeches must be cautiously employed. If we venture on general bleeding, the quantity of blood drawn should not exceed ten or twelve ounces, and, if necessary, this may again be abstracted in twelve or twenty-four hours. In most cases leeches to the temples, fore-head, and eyelids are preferable. One or more smart doses of calomel and colocynth, followed by salts and senna, should be given to open the bowels freely; if the tongue continues foul, a common dose of ipecacuan and tartar emetic may be useful, after which, laxatives and some mild diaphoretic. The free use of mercury is improper, but an alterative course, with remedies directed to the digestive organs, is very beneficial. We have no experience of turpentine. Mr. Mackenzie has sometimes derived very striking benefit from the precipitated carbonate of iron, after depletion and mercury had failed. Sulphate of quina might be tried with some hope of success. Counter-irritation by blistering, or the tartar-emetic ointment is of great service. Dry warmth, by means of several folds of old linen, heated at the fire, hung over the eye, and renewed frequently, is the only direct application that can at all times be safely used. Cold applications do harm; hot fomentations are not always harmless, especially if the parts are left wet and exposed afterwards.

Moderation and speedy removal of the fits of pain is of great importance. Beer recommends opium, rubbed to the consistence of a liniment, to be rubbed in round the orbit; but mercurial ointment with opium and extract of belladonna, or volatile liniment with laudanum, may be applied when the evening paroxysm is expected to recur, and repeated, if necessary during the night or day. Opium internally should be avoided unless the pains become very urgent, but much relief may be obtained by the other and milder narcotics. Our author has found a vinous solution of the murias hydrargyri with belladonna, a convenient form for exhibiting the latter medicine as a sedative, and the former as an alterative in this disease. The apparent causes of the occasions of pain, as, agitation, changes of temperature, &c. must be avoided.

Relapses are to be warded off, partly by constitutional, partly by local means. The former are in part medicinal, chiefly dietetical. A temperate diet, gentle aperients, promotion of the action of the kidneys by magnesia and soda water, or some mild aperient and diuretic mineral water, will be highly beneficial. Daily tepid sponging of the body followed by dry friction, pure country air, regular, continued and varied exercise, and the avoiding of sudden changes of temperature are all important. If the patient has long been accustomed to wine he may be allowed a small quantity of spirits and water.

"After an attack of gouty inflammation in the foot, we see the parts continue long tumid, weak, and morbidly sensible, while the most trifling accident, internal or external, is apt to produce a relapse. The same is observed in regard to the eye, only that in this organ we have the advantage of directly witnessing the exceedingly relaxed, varicose, and livid state of the blood-vessels, an indication of how much is wanting to restore the affected parts to their natural tone. Even after an acute attack of arthritic iritis is subdued, some counter-irritating means ought to be continued, such as a seton in the neck, and recourse should be had to the use of local applications of a tonic kind. As a means of this sort, the Germans are in the way of using small bags of dried aromatic herbs, suspended over the eye. The bags are made of old linen, and are quilted, so as to keep the herbs equally spread out. The aroma, constantly emanating from the herbs, imparts a permanent, pleasant, and useful stimulus to the debilitated blood-vessels and nerves. The best herbs for this purpose, are bruised chamomile flowers, sage, rosemary, marjoram, and the like, with or without the addition of a little powdered camphor. If the exhaled aroma reproduces redness of the eye or aversion to light, this will indicate that the proper time for the use of local stimuli has not yet arrived, and that they must be postponed. Friction round the orbit once or twice daily with alcohol, tinctura aromatica ammoniata, or the like, is another local preventive measure which is found of use. Even stimulants to the eye, as vinum opii and red precipitate salve, beginning these preparations in a dilute state, and gradually augmenting their strength, are found to abate the morbid sensibility of the eye, and thus render it less apt to suffer from the ordinary external as well as internal causes which produce inflammation. It must not be forgotten, however, that remedies of this kind, if used before the inflammation is completely subdued, will, as in every other species of iritis, produce the very worst effects." 457.

### V.—Choroiditis.

The choroid coat being completely hid from view and exercising only a subsidiary function, inflammation of it has scarcely attracted attention. In an early stage this is one of the least striking of the ophthalmiæ, and the disorganization which it produces when advanced, has either escaped notice, or not been referred to its true and legitimate cause.

"I have already had occasion to mention, that iritis is occasionally attended by inflammation of the choroid. Were we to adopt the common notion, that the iris is a continuation of that membrane, we might be led to conclude, that choroiditis and iritis should always go together. Perhaps, in some degree, this may still be the case. At the same time, from the arteries which nourish these two parts being quite distinct in their course and distribution, the idea of a separate iritis, and a separate choroiditis, is *a priori* rendered probable.

"For some time, the separate existence of choroiditis was with me rather a matter of speculation, and a conclusion from analogy, than a fact ascertained by observation. I am now convinced, however, that the choroid is sometimes the seat, almost quite independently, of inflammation; that in certain cases of ophthalmia, it is the focus of the disease, and that the neighbouring parts may be as little affected when that is the case, as the sclerotica is in iritis, or the iris in sclerotitis. That it is of importance to distinguish the disease which I am now about to describe, will appear very evident, when we consider its dangerous nature. Its symptoms, as we shall immediately see, are very different from those of any other ophthalmia; and although ultimately the whole eye may be involved by inflammation commencing in the choroid, yet choroiditis, in the early stage, exists without any signs of disease in the iris, and without any other effects upon the sclerotica and retina, than those which must necessarily arise from the pressure of an inflamed and swoln membrane, placed in contiguity with other membranes, more or less susceptible of suffering from that pressure. I consider choroiditis, therefore, as completely a primary and distinct disease." 458.

*Symptoms.* From the pressure outwards of the inflamed and tumefied choroid, the exterior tunics of the eye become attenuated, so that the choroid shews its dark colour through the sclerotica, which therefore appears blue or purplish. This is a remarkable, and often an early symptom. The degree of discolouration varies with the severity and duration of the attack. After a time the affected part protrudes, commonly on one side only, near the cornea, as if the corpus ciliare was the seat of the disease, and more frequently above or to the temporal side of the cornea than below or to its nasal side. The tumour may enlarge to the size of half a filbert, or more, when it is generally of deep blue colour, with varicose vessels running over it, and has been called *sclerotic staphyloma.* Several such tumors may surround the cornea. The front of the eye is not the only seat of this sclerotic, or rather choroid staphyloma. Scarpa twice met with staphyloma of the posterior hemisphere of the sclerotica in the dead body. In one case, that of a woman forty years of age, who had lost the vision of the affected eye some years before, in consequence of an obstinate painful ophthalmia, the vitreous humor was found converted into limpid water, the chrystalline somewhat yellowish, but not opaque; there was a deficiency of the nervous expansion of the retina within the cavity of the staphyloma; the choroid was very thin at this part, deprived of its natural colour, and its usual vascular net-work; and the sclerotica, particularly at the apex of the staphyloma, was so thin as scarcely to equal the thickness of writing paper.

There is no doubt that the vessels of the choroid are greatly enlarged; our author saw, in the hands of Professor Beer, a preparation in which the varices of an inflamed choroid were as large as small peas. But frequently the distention of the choroid and sclerotica is connected with an effusion of watery fluid between the choroid and retina. This Mr. Mackenzie has frequently had occasion to evacuate with a needle. If this is not done it accumulates and presses the retina before it, produces an absorption of the vitreous humour, and gathers the retina into a cord, which, stretching from the entrance of the optic nerve to behind the lens, is seen through the pupil, looking like a deep-seated cataract, or the advancing tumour of fungus hæmatodes. The visible arteries of the sclerotica are much enlarged, and ramify over the distended portion. Not unfrequently there is a patch of redness near the cornea, fed by one or more of these arteries greatly dilated. Sometimes the redness is confined to the upper part of the eyeball; there is scarcely ever any general redness.

The iris is not affected, but the pupil in almost every case undergoes a remarkable change of place. The iris is always narrowed towards the affected portion of the choroid, often the pupil is almost directly behind the edge of the cornea; it is most frequently displaced upwards, and upwards and outwards. Occasionally it continues small and immoveable, in other cases it is immoveable, but not dilated; in very severe cases it is greatly enlarged, the iris having nearly disappeared at that part of its circumference towards which the displacement of the pupil has happened. The pupil does not return to its place, even though the choroiditis is subdued. Opacity of the cornea, generally the edge nearest to the portion of affected choroid, is a frequent attendant on choroiditis. In other cases there are pretty extensive, but very irregular spots of whiteness. In some severe and old cases the cornea becomes almost altogether opaque, and, partaking in the staphylomatous degeneration of the neighbouring sclerotica, even undergoes a degeee of dilatation. From this affection of the cornea alone vision may be almost or altogether lost.

In consequence of choroiditis the eye may enlarge, and even protrude considerably from the orbit, when it is liable to suffer from external inflammation. If this inflammation runs high the conjunctiva becomes chemosed, puriform fluid is deposited behind the cornea or between its lamellæ, the eye bursts, continues to swell and protrude, assumes a fungous appearance, bleeds profusely, and produces such pain and deformity as to require extirpation.

Intolerance of light and epiphora are generally considerable. The pain varies much in different individuals. When the sclerotica is much distended, especially when suddenly so, there is much increase of redness, and severe, sometimes furious pain. Hemicrania is present, not strictly circum-orbital or nocturnal. Vision is variously affected. Dimness of sight is sometimes the first symptom, or hemiopia, all objects to one or other side of a perpendicular line, or above or below a horizontal line appearing dim, or all objects appearing confusedly and as if double, even when viewed with one eye. Total blindness may ensue when the choroid seems only partially affected, or a good share of vision may be retained when the whole choroid is evidently affected.

*Constitutional Symptoms.* The subjects of the disease are adults only, and chiefly those of strumous constitution. In the early stage of the disease, before distention brings on acute pain, the pulse is not affected; after the patient has suffered much, a cachectic state is apt to follow, with quick pulse, pale or sallow complexion, great irritability, and general weakness. The digestive organs are frequently much deranged, even from the very first.

*Remote and exciting Causes.* Want of exercise and of the open air—derangement of the stomach and bowels—over-use of the eyes in reading, sewing, &c.—exposure to too much heat and light, especially to the glare of hot fires, and to sudden changes from heat to cold—blows upon the eye.

*Prognosis.* Recovery is always slow, and if the disease has gone to any considerable length, it is scarcely ever complete, the vestiges being generally permanent, although the progress has been fairly checked. In many cases we are lucky if we arrest the disease, yet sometimes the cure proceeds to a degree beyond our expectation. Mr. Mackenzie gives a case in illustration of what we can sometimes effect.

*Treatment.* Profuse and repeated blood-letting is of the utmost importance in the early stage of choroiditis. A person not acquainted with the nature of the disease might be tempted, from the little appearance of inflammation, to apply merely a few leeches, when he should be opening the temporal artery, and removing a large quantity of blood. Mr. Mackenzie has known the blueness and distention of the sclerotica, which had continued unabated by leeches and other remedies, for many weeks, disappear suddenly and completely after the loss of twenty or thirty ounces of blood from the temple. Bleeding from the jugular vein, or from the arm is highly useful; and so is the application, every second day, of twenty-four or more leeches round the eye. In chronic cases we must not neglect the free and frequent use of leeches. Purgatives are of great service—calomel, as a cholagogue, followed by salts and senna should be repeated frequently during the treatment. We might naturally expect that mercury would be beneficial here, as in iritis, but Mr. Mackenzie, who has used it in all forms and doses, has not hitherto found much advantage from its employment. He still prescribes it because he has seen too few cases to enable him to pronounce definitively on its value, and because it does good in all other chronic inflammations of the eye. Turpentine has been lately tried by our author, but he has come to no conclusion respecting its efficacy. In one case, in which Mr. Mackenzie had made up his mind to extirpate the eye, it shrunk considerably under the use of iodine, while the sclerotica assumed much more of its natural whiteness. After due depletion, Mr. M. has seen great good from the precipitated carbonate of iron and sulphate of quina. Counter-irritation, best obtained by a tartar-emetic eruption between the shoulders, is very useful. Puncturing the sclerotica and choroid, so as to evacuate the aqueous fluid collected between the latter tunic and the retina, is of much importance in the chronic stage of the disease, for Mr. Mackenzie has not ventured to try it in the acute. The operation is performed by thrusting a broad cataract-needle to the depth of an eighth of an inch, not in the direction of the lens, but towards the centre of the vitreous humor. The operation gives great relief, and may be repeated every eight days, or at longer intervals, according to the state of the eye.

### VI. RETINITIS.

"It is easy to understand that the internal inflammations of the eye may arise sometimes in one texture, and at other times in another; that in one case the bloodvessels of the retina shall be first affected, in another, those of the choroid, in a third, those of the iris. The point of origination will depend on the natural constitution of the organ, and the manner of action of the exciting cause. Even from birth, the eye varies much in different individuals, one or other texture appearing to be congenitally weaker or stronger than the others, so that the same exciting cause, operating on a number of persons, shall produce in one, inflammation of the conjunctiva; in another, sclerotitis; in a third, iritis; in a fourth, inflammation of the retina. On the other hand, the nature of the cause leads in one case to external, in another, to internal ophthalmia. Cold, operating on the eye, will bring on inflammation of the conjunctiva or sclerotica, while the sudden and direct reflection of a strong light into the eye will be apt to produce an inflammation of which the retina is likely to be the focus. The inflammatory action, however, is seldom, if ever confined to the part first affected. We have already seen how inflammation, originating in the iris, spreads to the sclerotica, and to the choroid; and how choroiditis affects the textures both within and without the choroid. In the same way, inflammation commencing in the retina is likely to spread inwards to the vitreous humour, to the capsule of the lens, and to the lens itself, all which parts are fed by branches from the central artery of the retina; and outwards, to the choroid and iris, to the sclerotica and cornea, and to the conjunctiva. Thus an inflammation of the whole eyeball may arise from a very limited point of origin.

Nor is this a fanciful picture of disease. Although a retinitis, ending in general ophthalmitis, and arising from causes of very limited and transient action, is rare; yet it occasionally occurs, especially after long continued straining of the sight in the examination of very small, perhaps microscopical objects, under a strong light, reflected into the eye, either immediately from the object of examination, or from a speculum.

In such cases, however, there are commonly certain predisposing causes, which ought not to escape observation; such as plethora in and near the organ of vision.

Unexpected and vivid flashes of lightning sometimes excite inflammation of the retina, and this disease has frequently been excited by imprudently viewing an eclipse of the sun. Prisoners, who have been long confined to the darkness of a dungeon, have been seized with inflammation of the retina on being brought suddenly forth into the full glare of day. Travelling over a long tract of country covered with snow, has been known to produce the same effect. Saint-Yves notices the case of a man who became blind in consequence of going too close to the light and heat of a strong fire, in attempting to tie a string to a fowl, turning on the spit; and another, of a workman in the mint, who lost his sight from the brilliant flashing to which he was exposed, while pouring metal into a red-hot crucible. Both of these accidents were probably owing to retinitis.

The Esquimaux, who inhabit Hudson's Bay, are well aware of the loss of vision which arises from exposing the eyes to the constant view of a country covered with snow. They make use of a kind of preservers, which they term snow-eyes. These consist of two pieces of wood or ivory, so formed as to fit the eyes, which they completely cover, and are fastened behind the head. Each piece presents a narrow slit, through which every thing is distinctly seen. This invention preserves them from the snow-blindness, which is apt to be occasioned by the strong reflection of the sun's rays; and which, it is probable, is the effect of inflammation excited in the retina.*

* "These instruments also increase the powers of vision, so that the Esquimaux are so accustomed to their use, that when they are desirous of viewing any thing at a distance, they mechanically apply them to their eyes. Different accounts are given of the slit or slits in these instruments, for some tell us there is only one in each eye-piece, and that it is long and narrow, while others say that there are two, about a quarter of an inch long. This is probably regulated by the fancy of the wearer."

Blinding persons by producing retinitis was, and still is, in some countries, a mode of punishment. The person is compelled to look steadily on a concave mirror of polished steel, held opposite to the sun. This would excite speedy inflammation of the retina, and certainly end in a greater or less degree of insensibility to light. Some such method must be employed in India at this day, as many of the native princes, who have been condemned to the loss of sight by the jealousy of their rivals, but are suffered to live in a state of captivity, are said to have no appearance, at a little distance, of being blind." 467.

Chronic cases of retinitis are not unfrequent, under the designation of weakness of sight; there are morbid sensibility to light and slight obscurity of vision, followed by gradual contraction of the pupil, immobility of the iris, and amaurosis. Watchmakers, jewellers, and those who spend much time in reading and writing are liable to be affected. Stimulant and tonic treatment is injurious; leeches round the eye beneficial. In strumous ophthalmia, it is conjunctivitis and not retinitis which causes the excessive intolerance of light.

*Symptoms of Acute Retinitis.* The patient first complains of a general feeling of pressure and tension of the whole eyeball, succeeded by obtuse, deep-seated, pulsative pain, which seems to increase every moment, and soon extends to the eyebrow and cranium. The power of vision is already sensibly diminished, and every hour becomes more and more feeble, the pupil has lost its glancing blackness, and has become much contracted. At last it completely closes, the iris having reached its greatest possible degree of expansion. Long before the pupil is closed, the sensibility of the retina seems extinct, and yet when the pupil *has* closed, the patient experiences a troublesome sensation of fiery spectra, with every oscillation of the internal blood-vessels of the eye. During these changes the iris becomes discoloured, greenish or reddish according to its original hue. The anterior chamber is strikingly diminished in size by the advance of the iris towards the cornea, during which the whole sclerotica is rose red. Some time after this the conjunctiva presents a pretty thick net-work of blood-vessels, and the cornea loses much of its natural lustre, without becoming absolutely opaque. Now there is severe inflammation, fever, with almost maddening headache. Sometimes, during this period of the disease, the pupil does not completely close, but it is cloudy, and on looking at it through a magnifying glass, is seen to be reddish-grey, while the power of vision is totally lost. So severe are the fever and headache that retinitis is often mistaken for phrenitis. The pain of the eye now becomes unequal, and though still pulsative is attended with a feeling of cold and weight in the part. Shiverings take place, and suddenly there appears a quantity of pus gravitating to the bottom of the anterior chamber. It may accumulate to such a degree, especially in neglected cases, that the cornea projects and at last gives way under insufferable pain; the eye then collapses, and the pain gradually subsides. If the pupil has not quite closed by the end of the first stage, we see, just where the hypopium begins, fine whitish filaments of lymph, shooting from the edge of the cornea towards its centre. Viewed through a good lens these have the appearance of a delicate cobweb. After the pus has covered the pupil and remained, perhaps, long unabsorbed, this pseudo-membrane becomes whitish-yellow, from little particles of the pus lodging in the interstices, and sometimes a single piece of what seems to be thickened purulent matter, attached to this membrane, projects through the pupil, intimately connected with the pupillary edge of the iris. If the pupil has closed completely in the first stage this spurious cataract cannot, of course, be observed.

*Prognosis.* If proper treatment be commenced before the pupil is much contracted, the prognosis is not unfavourable; but if vision seems already extinguished, it is extremely unfavourable. If the pupil is once closed, even before the retina appears to have become insensible, there is no longer any hope of preserving sight; for even should the pupil re-open in some degree, yet it remains small and motionless, and the eye is still blind. If retinitis is neglected or mistreated in the commencement, it proceeds rapidly to a dangerous inflammation of the whole eyeball. In the second stage the prognosis is always bad, for before the disease has advanced so far, vision is irretrievably lost. All that can be done is to endeavour to save the form of the eye, by limiting the suppuration as much as possible. If the disease has gone on to a complete ophthalmitis, attended with chemosis, we may not even be able to do this.

"*Treatment.* Complete rest of the eyes and of the whole body, darkness, abstinence, and active depletion, followed by the rapid introduction of mercury into the system, are the means to be depended upon in the first stage of retinitis. Copious blood-letting from the arm is to be immediately followed by a plentiful application of leeches round the eye. Should the pain of the eye and head still continue, the jugular vein or temporal artery ought to be opened, and a considerable quantity of blood abstracted.

Calomel with opium ought to be given in frequent doses, till the mouth is affected.

Belladonna is to be applied in the usual way.

In the second stage, the preservation of sight is out of the question. A warm emollient poultice is to be laid over the eyelids. If only a small quantity of matter be present in the anterior chamber, we must on no account let ourselves be induced by that to open the cornea, for the purpose of evacuating it; but trust to the sorbefacient effect of the mercury, assisted by blisters behind the ears or on the back of the neck. Beer recommends the eye in that state to be touched repeatedly in the course of the day with vinum opii, by the careful use of which, in combination with the internal employment of opium and sometimes of cinchona, he had seen collections of pus in the anterior chamber completely disappear. Should the hypopium increase, so that the anterior chamber is filled, we cannot trust to its absorption, but must give exit to the matter by opening the cornea with the extraction knife. In such circumstances, the natural appearance of the cornea and iris is completely lost, the eyeball sometimes remaining flattened in the situation of the cornea, while in other cases it becomes staphylomatous." 470.

### VII. AQUO-CAPSULITIS.

By this term is meant inflammation of the cartilaginous membrane lining the internal surface of the cornea. When inflamed it becomes more or less opaque, there is a muddiness in the anterior chamber, and occasionally an appearance as if the eye were unusually full and prominent, arising from an increase in the quantity of the aqueous humour. In more severe cases coagulable lymph is effused, and if the iris be inflamed at the same time, this effusion may unite it with the cornea. Besides the diffused muddiness there are often present one or more milk-white spots on the internal surface of the cornea, giving it a mottled appearance, and forming the most characteristic mark. Mr. Wardrop has accurately described their more opaque central points as surrounded by a kind of disk, resembling what is called the eye of a pebble. In one of two cases in which Mr. Mackenzie distinctly observed this appearance, the spots appeared and disappeared at different points of the internal surface of the cornea even in the space of a few hours, so that the patient saw worse in the morning, when most of the spots were observed, and better towards evening, when those at the upper part of the cornea had greatly diminished. As in iritis, there is a circular zone of minute vessels seen on the anterior part of the sclerotica; sometimes one or more distinct blood-vessels are seen traversing the inflamed membrane; some vessels of the conjunctiva also are frequently enlarged, appearing as insulated trunks. The vessels on the white of the eye are of bright red colour, and gradually assume a more crimson hue, as the symptoms subside. Sometimes there is epiphora, but generally the patient suffers very little from exposure to light. Vision is more or less dim, and there is a particular sensation of fulness and distention of the eyeball, with dull aching pain, generally in the forehead, occasionally also in the back of head; symptoms, which Mr. Wardrop avers to be instantly and permanently relieved by evacuating the aqueous humour.

The constitutional symptoms vary much in their degree of severity. Sometimes there is considerable pyrexia; in other cases, the disease assumes from the first a chronic form, and, after a certain period, participates in any peculiarity of the patient's constitution, and becomes thereby modified. During the inflammatory symptoms there is usually so much muddiness of the anterior chamber, that no distinct portions of lymph, unless of large size, can be distinguished. When the turbidness goes off, flakes of lymph may sometimes be perceived; at others, the whole surface of the inflamed membrane is covered by a thin layer of it. Sometimes the lymph floats like a thick cloud in the anterior chamber; in other cases it is deposited in streaks, of a reticulated appearance; in others, it resembles a purulent fluid. If the lymph is not afterwards absorbed it may become organized, and not unfrequently red vessels can be seen ramifying through it. This is more frequent than a red vessel or vessels running along the internal surface of the cornea without any effusion of lymph.

"*Treatment.* Little else is known regarding the effects of remedies in this rare ophthalmia, than what is mentioned by Mr. Wardrop, in his paper on Evacuation of the Aqueous Humour, in the fourth volume of the Medico-Chirurgical Transactions. In the cases there recorded, benefit appears to have been derived from cupping the temples, purging, fomenting, and the application of such stimulants as murias and nitras hydrargyri in solution, red precipitate salve, and sulphuric ether. Mr. Wardrop, however, places most reliance on the evacuation of the aqueous humour, stating that there is no inflammation of the eye, where so much benefit is derived from that operation, as when the disease affects the internal layer of the cornea. He had never found it fail in procuring immediate relief of the pain of the head, and instantaneous restoration of the transparency of the anterior chamber.

The opening through the cornea, by which the aqueous humour is to be discharged, may be made with any of the knives commonly used for extracting the cataract, or with a broad iris-knife. It is sufficient that the point of the instrument be introduced so that it makes a puncture into the anterior chamber; this should be done near the junction of the cornea and sclerotica, at any part of the circumference. When the knife has penetrated into the anterior chamber, it may be withdrawn a little, and the blade turned on its axis, when the aqueous humour will readily escape. It is better not to remove the instrument altogether, till the fluid is observed to be discharged; for if the incision be not sufficiently large, and the knife taken away before the aqueous humour flows out, the elasticity of the cornea closes the wound, and either hinders the evacuation from being so sudden, and consequently so efficacious, or the closure of the wound entirely prevents its escape. The operation, therefore, which is necessary to discharge the aqueous humour, is merely the first step of the section of the cornea, made in extracting the cataract, or what is called the *punctuation*.

The chief difficulty in performing the operation, arises from the pain occasioned by the necessary pressure on the eyeball, whilst keeping open the eyelids; but until a sufficient portion of the cornea is brought into view, and the movements of the eye completely under the management of the operator, the introduction of the knife should not be attempted. The upper lid should be elevated by the fingers of the assistant, or by Pellier's speculum; while the operator, with the fore and middle fingers of the hand which does not hold the knife presses down the lower lid, and applies their points over its edge, in such a manner that they touch the eyeball, and can apply any degree of pressure upon it which may be necessary. After the assistant raises the upper lid, the patient should be directed to look downwards; and then the assistant employs a sufficient pressure, to keep the eye in that position.

The operator now makes the puncture; but as the patient is very apt to start when he first finds the instrument coming in contact with his eye, it is useful merely to touch the cornea repeatedly with the back of the knife till all risk of starting is over; and as soon as its extremity rests on the part where the puncture is to be made, the knife may readily be raised on its point, and thrust into the anterior chamber.*

It is probable that a variety of other remedies besides those mentioned by Mr. Wardrop might be useful in aquo-capsulitis; especially cinchona, turpentine, and mercury. Of these, however, nothing can be said from experience." 473.

### VIII. Inflammation of the Crystalline Lens and Capsule.

Common lenticular cataract appears to be a consequence of the impeded nutrition o' advanced life, whilst opacities of the capsule are probably always the result of inflammation. Capsular and capsulo-lenticular cataracts are rarely seen till the inflammation has subsided, but Mr. M. confirms the accuracy of Professor Walther's description.

Inflammation of the capsule generally occurs about the middle of life, and in slightly cachectic subjects, but our author has more than once seen it in young children. It occurs oftener in light eyes than dark, and the iris becomes a little darker, the pupil somewhat oval or irregular. The motions of the iris are at first free, but subsequently sluggish and very limited. The pupil is smaller than natural, and there is usually a black rim of irregular breadth round its edge, arising from the pigmentum nigrum of the posterior surface of the iris coming into view. Along with these symptoms, a number of red vessels appear in the pupil itself, the largest of which are visible

---

* "Medico-Chirurgical Transactions, Vol. iv. p. 153. London, 1813."

to the naked eye, but the majority are only discernible by the aid of a magnifying glass. This should be of short focus, and to have the pupil as large as possible, the other eye should be closed during the examination, and a little of a filtered solution of extract of belladonna dropped on the affected one an hour previously.

The red vessels observed in the pupil always form a sort of vascular wreath, situated about a quarter of a line's distance from the pupillary edge of the iris; this wreath forms a concentric circle within the pupil, and consists of a number of vascular arches. To it numerous vessels run in a radiated form from the circumference of the capsule; others, but not always, seem to extend from the pigmentum of the iris; in other cases, vessels seem to be prolonged from the capsule into the posterior surface of the iris. Those running from the iris to the capsule, always arise at a little distance from the edge of the pupil, on the posterior surface of the iris, so that nearly a line's breadth next the pupillary edge is free from these vascular sproutings. From the vascular wreath, vessels are seen spreading towards the centre of the anterior capsule, again forming clusters and arches. No doubt all the vessels at different parts of the capsule are continuous. Posterior to the red vessels seen in the capsule, there appears in some cases a net-work of more delicate vessels, which seem to be seated in the lens itself. The larger evidently come, says Professor Walther, from its posterior surface directly forwards, and then divide into branches. He has repeatedly seen these vessels in the lens, and they constitute a very beautiful phenomenon. He thinks that their existence is morbid. It would appear that all inflammations of the lens begin in the capsule, a fact which Professor W. considers as analogous to the spread of inflammation to the capsule from the ciliary processes or from the iris.

At the apparent termination of several of the vessels in the capsule there are distinctly perceived little knots of a whitish grey semi-transparent substance, evidently coagulable lymph, and explaining the manner in which opacities of these parts are formed. The anterior hemisphere of the capsule, where the vessels are very numerous, sometimes assumes a peculiar velvety or flocculent appearance, and in one or more spots of its extent presents a grey or brownish colour. These brownish spots appear in some instances to be merely effused lymph; but in other cases they probably owe their origin to the iris having been united to the capsule by partial adhesions, which being separated in one way or other, part of the pigment of the iris has remained adherent to the anterior surface of the capsule.

Where inflammation of the lens and capsule is severe, vision is indistinct and confused, particularly when the eye is directed towards distant objects; those nearer are seen as through a fine gauze which does not seem red, nor are the objects tinged so. This ophthalmia is always chronic, proceeding slowly, and attended with little pain. When this does exist it is seated at the bottom of the orbit, in the forehead, or in the crown of the head. When the disease has continued long the blood-vessels in the lens and capsule become varicose, and permanently remain so. In one case Mr. M. has seen incomplete amaurosis, with tremulous iris, follow this disease. Effusion of fluid between the lens and capsule, and dissolution of the former are not unfrequent consequences; and, in other instances, the inflammation appears to run on to suppuration, for in the variety of cataract called the *cataracta*

*cum bursa* the opaque state of the lens and capsule is combined with a cyst contained within the capsule and filled with pus. The causes of this ophthalmia have not been sufficiently investigated; in one case Mr. Mackenzie observed it in the right eye of a keen sportsman. Inflammation of the lens and capsule approaches nearer to iritis than any other ophthalmia, but is much less severe and much less under the influence of treatment.

In the early stage of the disease depletion, counter-irritation, and alteratives seem indicated, in the latter stages tonics. With our author, this has proved the most obstinate of ophthalmiæ, even mercury appearing to have scarcely any power.

### IX. Inflammation of the Hyaloid Membrane.

Though the morbid states with which we meet in the vitreous humour naturally give rise to the supposition, that its investing membrane must occasionally suffer from inflammation, and though other considerations would lead to the same conclusions, yet such inflammation has not been observed with sufficient accuracy to warrant description.

### X. Traumatic Ophthalmiæ.

Each texture of the eye has been shewn to suffer in its own way from inflammation, excited without any mechanical or chemical injury. The inflammation, on the other hand, excited by such injury, may attack one or several of the textures of the eye; when thus excited the ophthalmia imitates the ophthalmiæ already described. Traumatic iritis very closely resembles the rheumatic; the cornea, by traumatic inflammation, is rendered opaque, or becomes affected with onyx or with ulceration; and so on. Upon this hint we may act in our treatment; traumatic iritis, for instance, is to be treated exactly as the syphilitic or rheumatic; puriform traumatic inflammation of the conjunctiva, precisely like catarrhal ophthalmia. The consideration of the former varieties of ophthalmia will teach us to understand perfectly the effects of mechanical and chemical injuries, and though the symptoms may vary very greatly in severity, yet the inflammation of each texture presents, under whatever circumstances, the same essential phenomena.

The most important general rule in the treatment of the traumatic ophthalmiæ is, that we should be upon our guard against effects that may be produced, but are not yet present, and against effects implicating the interior structure of the organ, though the injury seems to be superficial. Our treatment must be preventive; we must not wait till severe sclerotitis sets in, but bleed from the moment of a severe injury—nor till the pupil is evidently closing, but apply belladonna to prevent it—nor till the iris grows discoloured or lymph is effused into the pupil, but put the patient on calomel and opium, if we apprehend from the nature of the injury that iritis is likely to ensue. We sometimes meet with severe sympathetic inflammation in the eye which has not received the injury. It must be observed that, after all the other symptoms of severe traumatic inflammation of the eye have been removed by depletion, &c. a very troublesome and obstinate intolerance of light, with epiphora, is apt to remain, apparently from continued and now habitual excessive activity in the lids and lachrymal gland. In such cases, in addition to the usual remedies for epiphora, Mr. Mackenzie has derived advantage from the internal use of the extract of stramonium.

### XI. COMPOUND OPHTHALMIÆ.

Strictly speaking, few ophthalmiæ are absolutely simple. Many are strikingly compound, as the catarrho-rheumatic. Strumo-catarrhal ophthalmia is also very common; and we find phlyctenular conjunctivitis with strumous iritis, strumous corneitis with iritis, and many other compound ophthalmiæ. The treatment of such cases will of course consist in the combined use of the means adapted to the separate or simple ophthalmiæ. Thus, in strumo-catarrhal cases, we require the treatment necessary for strumous ophthalmia in combination with that for catarrhal conjunctivitis, and so on.

### XII. INTERMITTENT OPHTHALMIÆ.

"Although several interesting cases have been recorded of ophthalmiæ recurring in the same individual after longer or shorter intervals of time, yet I doubt whether there is sufficient ground to admit the existence of any disease of this kind so regularly periodic in its accession, as to warrant the appellation of *intermittent ophthalmia.* The pain which attends many of the ophthalmiæ, is undoubtedly subject to regular nocturnal exacerbations, but this does not entitle these diseases to the appellation of intermittent. By an intermittent or periodical ophthalmia, I should understand one which recurred with considerable regularity at intervals of weeks or months, and apparently not from accident, but from concatenation with the revolutions of time; whereas, if we examine the cases which are recorded as being of this kind, we shall find that they are nothing more than instances of some particular ophthalmia recurring more or less frequently in the same individual, in consequence of his repeatedly exposing himself to the same, or to some similar exciting cause. The strumous ophthalmia, being that which is most apt to be renewed on slight exposures, will also more frequently than any other inflammatory disease of the eye appear to be periodic. The rheumatic, catarrho-rheumatic, and catarrhal will also be subject, from their ready occurrence in eyes once affected with them, to the same suspicion. I have frequently treated patients who at intervals of three or four months, or once a year nearly about the same season for several successive years, had suffered an attack of rheumatic iritis; but in every case of this kind, I have been able to trace the return of the disease to some new imprudence. In arthritic inflammation of the eyes, the periodic tendency will also appear to be very decided, for every attack of that sort leaves the eyes worse than before, and with a strong disposition to suffer again from renewed causes of excitement.

These remarks, will, I think, be confirmed by a careful perusal of the interesting narratives of Dr. Curry and Dr. Bostock, both of whom had suffered from repeated attacks of severe ophthalmiæ." 481.

We have now arrived at the limits which we proposed to ourselves at the commencement of this article. The subject of the ophthalmiæ is concluded, and though Mr. Mackenzie proceeds in his next chapter to consider the diseases consequent on the ophthalmiæ, we cannot follow him at present. We have been careful to give Mr. Mackenzie's sentiments on the affections rescribed in this and the preceding article, precisely yet fully; for, as we before remarked, there is scarcely a practitioner who will not, at some time or other, and with more or less frequency, be called upon to treat them. We need scarcely remark how serious the consequences of ignorance may be, not only to the patient but to the practitioner himself. It is because this subject is one of such general, such vital importance, that we have dwelt on it so long. We have offered no critical remarks, for our object has been to communicate the opinions and practice of an experienced ophthalmologist to those who stand in need of such information, and whose *res angusta domi*, will not permit them to purchase the work itself. We do not imagine that Mr. Mackenzie is right on all points: were he so he would be little less than a phenomenon. Neither do we consider his book a vast fund of originality, in which every page is redolent with discoveries. A great part of its contents, nay, by far the greater part, will be found in other authors on the Diseases of the Eye, in the highly valuable volumes of Travers, Saunders, Guthrie, Lawrence, &c. But the divisions of ophthalmic surgery are arranged with more precision, and the doctrines laid down with more method and order in this than in most other works; and as it suits our purpose we make use of it. From time to time and on various occasions we shall select separate chapters or sections for analysis. We again conclude by recommending all who can afford it to purchase this very useful treatise.

